# Deep GRU-CNN Model for COVID-19 Detection From Chest X-Rays Data

**DOI:** 10.1109/ACCESS.2021.3077592

**Published:** 2021-05-05

**Authors:** Pir Masoom Shah, Faizan Ullah, Dilawar Shah, Abdullah Gani, Carsten Maple, Yulin Wang, Shahid, Mohammad Abrar, Saif Ul Islam

**Affiliations:** Department of Computer ScienceBacha Khan University Charsadda 24000 Pakistan; Faculty of Computer Science and Information TechnologyUniversity of Malaya 37447 Kuala Lumpur 50603 Malaysia; Faculty of Computing and InformaticsUniversity Malaysia Sabah Labuan 88400 Malaysia; Secure Cyber Systems Research Group, WMGUniversity of Warwick 2707 Coventry CV4 7AL U.K.; Alan Turing Institute London NW1 2DB U.K.; School of Computer ScienceWuhan University 12390 Wuhan 430072 China; Department of Computer ScienceMohi-ud-Din Islamic University 66933 Nerian Sharif 12080 Pakistan; Department of Computer ScienceInstitute of Space Technology 66753 Islamabad 44000 Pakistan

**Keywords:** Medical data, deep learning, CNN, GRU, COVID-19, chest X-rays

## Abstract

In the current era, data is growing exponentially due to advancements in smart devices. Data scientists apply a variety of learning-based techniques to identify underlying patterns in the medical data to address various health-related issues. In this context, automated disease detection has now become a central concern in medical science. Such approaches can reduce the mortality rate through accurate and timely diagnosis. COVID-19 is a modern virus that has spread all over the world and is affecting millions of people. Many countries are facing a shortage of testing kits, vaccines, and other resources due to significant and rapid growth in cases. In order to accelerate the testing process, scientists around the world have sought to create novel methods for the detection of the virus. In this paper, we propose a hybrid deep learning model based on a convolutional neural network (CNN) and gated recurrent unit (GRU) to detect the viral disease from chest X-rays (CXRs). In the proposed model, a CNN is used to extract features, and a GRU is used as a classifier. The model has been trained on 424 CXR images with 3 classes (COVID-19, Pneumonia, and Normal). The proposed model achieves encouraging results of 0.96, 0.96, and 0.95 in terms of precision, recall, and f1-score, respectively. These findings indicate how deep learning can significantly contribute to the early detection of COVID-19 in patients through the analysis of X-ray scans. Such indications can pave the way to mitigate the impact of the disease. We believe that this model can be an effective tool for medical practitioners for early diagnosis.

## I. Introduction

Artificial Intelligence (AI) applications for data analysis have revolutionized the medical field by achieving human-level accuracy in medical image classification [1]. Coronavirus disease or COVID-19 is a new type of contagious disease caused by a novel strain of flu virus. According to the world health organization (WHO), the first case of COVID-19 was first reported in the Chinese province Wuhan in December 2019 [2]. Coronavirus is recognised as the biggest global challenge in the 21st-century so far [3], [4]. On March 11, 2020 World Health Organization (WHO) declared the novel COVID-19 as a pandemic [5], [6].

Like other infectious diseases in the family of coronavirus, such as Middle East respiratory syndrome (MERS) and Severe Acute Respiratory Syndrome (SARS), COVID-19 infects the main respiratory organs of the human body [7], [8]. A patient infected with COVID-19 experiences symptoms such as coughing, fever, sore throat, tiredness, loss of taste and smell [9]. In many cases, infected patients have difficulty in breathing, kidney failure and chest pain, which may result in death [10]. Millions of people have lost their lives worldwide [11]. A number of pharmaceutical companies have achieved success in developing COVID-19 vaccines and numerous trials on humans have been completed or are in progress globally.

The detection of the infected people is a high priority in the battle to conquer this pandemic. According to the Chinese government, the detection of COVID-19 through gene sequencing can be performed using Reverse Transcription Polymerase Chain Reaction (RT-PCR) [12], [13]. Typically, this procedure requires four to six hours to achieve a complete result. Since this disease has spread over a large population, it becomes difficult for a testing laboratory to entertain a large number of tests and provide timely diagnosis [14]. Many patients remain undiagnosed and become a source of spreading the virus. Given the severity of the problem, there is a need for developing fully automated and time-efficient systems.

The early detection of COVID-19 can reduce the spread, and hospital load [15]. As discussed earlier, the RT-PCR testing producer has limitations. As such, the research community is investigating alternative solutions. X-ray and Computed Tomography (CTs) are two medical imaging techniques that have proved effective in detecting lung-related diseases. These techniques have also achieved promising results in the visualization of COVID-19 lung infection [16]. However, when a radiologist or other medical practitioner views the X-ray image, it is possible they may miss early signs of COVID-19. Further, there is a possibility that different experts may come up with a different conclusions.

Recently there has been an increase in medical diagnoses through automated processes. Machine learning algorithms have shown higher accuracy in the detection of several diseases than domain experts. In recent years, Chexnet [17] beat human vision in terms of thoracic disease classification through chest X-rays. Further, PirShah [18] applied CNN on Magnetic Resonance Image (MRI) data to detect Parkinson’s disease and achieved state of the art accuracy. In the same way, different researchers have attempted to use CNN for COVID-19 detection from chest X-rays [19]. Ozturk *et al.* [20] elaborated the importance of the early recovery of the COVID-19 positive patients. They have discussed methods for the detection of the virus. They find that detection of COVID-19 in patients through Computed Tomography and X-rays has been discussed in detail, and that these are useful for timely detection. The authors claim that detection is first performed in binary decision, that is COVID or Non-COVID. In the second approach, the detection is a multi-class classification which is COVID vs Non-COVID vs pneumonia. They have used a data set of 125 X-ray images for their experiments and have obtained an accuracy of 98% for binary while 87% for the detection of COVID-19 disease in a multi-class setting. Inspired by their research, there is a need for a more robust model to diagnose COVID-19 from chest X-rays. Nguyen *et al.* developed a hybrid model combining GRU and CNN for handwritten digit recognition which achieved encouraging results in terms of accuracy [21]. We adopted the same strategy of combining CNN and GRU for the detection of COVID-19. CNN is used for feature extraction, while GRU is used as a fully connected layer. Since COVID-19 is a novel disease there is limited data publicly available for experiments. The data set used for this study is obtained from two different sources. COVID -19 infection Xrays are obtained from [22] while pneumonia and normal images are acquired from the Kaggle repository [23]. Previously many deep learning models have been applied to COVID-19 datasets. However, the limited size of datasets presents a challenge. In particular, CNN suffers from a weight vanishing problem with limited data. To address this issue, GRU and LSTM have been previously deployed. We adopt propose a similar framework. To the best of our knowledge, it is the very first attempt to use GRU for COVID-19. The main contributions in this paper are as follows:

1) Proposition of a hybrid deep learning model based on convolutional neural network (CNN) and gated recurrent unit GRU) for diagnosing COVID-19 from chest X-rays (CXRs).
2) We utilised a CNN with ten convolutional layers and five max-pooling layers for feature extraction from chest X-ray images.
3) To overcome the weight vanishing problem with limited data, we used a GRU for classification.
4) We visualize the decision of the proposed model on X-rays using CAM.

This paper is organized as follows: In section II, related studies are reviewed. Section III presents the building blocks of a convolutional neural network, while section IV discusses the proposed technique in detail. Performance evaluation of the proposed technique is presented in section V before section VI concludes the paper.

## II. Related Work

In recent years many researchers have proposed AI algorithms to address medical related issues. Algorithms based on deep learning are now being used in several domains [36]–[38]. By utilizing Convolution Neural Network (CNNs) researchers are able to achieve promising results in the field of medicine, including brain tumor segmentation, breast cancer detection, thoracic disease classification in X-ray images and so on. Several strategies for disease detection from biomedical imaging data have already been proposed by various scholars. Sharma and Miglani [39] highlighted the key and future challenges of medical image processing. Lee and Fujita [40], describes a number of studies for the detection of various diseases through the use of deep learning algorithms. Cho *et al.* [30] proposed a deep learning algorithm for dermatologist-level classification of malignant lip diseases using deep convolution neural network. The author trained the ResNet model on a dataset of 1629 clinical images. The performance of the proposed method was evaluated using different sets of images having 281 and 344 instances. The proposed model is compared with 44 participants for classification purposes. Qu *et al.* [31] suggested a novel 3D dense separated convolution (3D-DSC) module for volumetric medical image analysis. In this study the traditional 3D convolutional kernels are replaced with 3D-DSC. The 3D-DSC architecture is assembled using a series of densely attached 1D filters.

Hashmi *et al.* [32] used deep transfer learning techniques for efficient pneumonia detection in chest X-ray images. A novel ensemble approach based on a weighted classifier is introduced. The proposed model merges the prediction results of a weighted classifier from the state of art deep learning algorithms. Son *et al.* [33] presented a deep learning algorithm for validation and development for detecting multiple anomalies findings in retinal fundus images. Baltruschat *et al.* [27] developed a deep learning approaches comparison for multi-label chest X-rays classification. In this study transfer learning with and without fine tuning is utilized.

In Xue *et al.* [26], X-ray images of chest are passed through a process of evaluation, called optimization of scan lines, to remove all parts of the body to minimize error during diagnosis. They address an issue that traditional methods for image restoration suffer when finding locally optimal solutions rather than global, thereby achieving low accuracy results. They also address the issue of high computational load requirements in 4D CT picture registration.

Nasullah *et al.* [28] developed a modified technique based on a pair of deep, customised three dimensional mixed link networks (CMixNet) for classification and detection of lung cancer. Nodules of lungs were classified using a gradient boosting machine (GBM) by utilizing extracted features from the CMixNet module. The results of deep learning nodule based classification were compared with several factors including patient family history, history of smoking, age, clinical biomarkers, location and size of detected nodule. Yao *et al.* [24] modeled two different algorithms, long short term memory network and DenseNet, to extract anomalies and dependency. In this study author suggested a two stage end-to-end neural network algorithms that merge a densely attach picture encoder to a recurrent neural network decoder.

Recently several researchers have worked on classification of pneumonia. Khatri *et al.* [34] suggested to utilize earth movers distance (EMD) algorithm to classify non-infected and infected lungs. Preprocessing is performed on the source image to remove all non-lung areas. The preprocessed image is then resized, normalized by intensity so that a set of uniform shape/size of each lung is obtained. Stephen *et al.* [29] develop an efficient deep learning approach for classification pneumonia. This study utilizes a CNN model to train for the detection and classification of pneumonia from chest X-rays dataset. To overcome the issue of overfitting and enhance generalization of the model, several data augmentation techniques are used to enhance the quality and size of the dataset. Goyal and Arora [10] utilize a convolution neural network algorithm to extract important features and perform classification of pneumonia and COVID-19. The dataset used in this study consisted of 748 images having three different class types. The classes are bacterial pneumonia, normal and COVID-19. Three different deep learning algorithms Restnet50, VGG16 and VGG19 were applied to perform classification. Rajaraman *et al. [17]* make an effort to explain the performance of modified CNN to classify pneumonia and also detect difference between viral and bacterial disease in pediatric CXRs. Classification of pneumonia from CXRs is a difficult job due to the presence of huge number of variables that are extraneous to pneumonia diagnosis.

The early detection of COVID-19 is essential for the timely isolation of patients to prevent spreading of the virus. In practice many methods have been slow and costly, therefore automatic detection is required. Detection of COVID-19 from X-ray images has been performed by Apostolopoulos et al in [41]. They utilized two datasets with 1427 images and 1442 images. These datasets are collected from publicly available repositories. Accuracy, sensitivity and specificity of the system using deep learning with transfer learning is 96%, 98% and 96% respectively. According to the authors, detection of COVID-19 via X-rays is a useful addition to the traditional testing methods.

Abdulkareem *et al.* [42] suggested a model based on the Internet of Things (IoT) and Machine Learning (ML) to diagnose COVID-19 patients in a smart hospital. The author suggested the use of ML to analyze laboratory findings can improve the accuracy rate of diagnosis (classification). Three different machine learning techniques, namely Random Forest (RF), Support Vector Machine (SVM) and Naive Bayes (NB), were utilized on a public dataset. The authors claim that they achieved up to 95% accuracy using the Support Vector Machine. Le *et al.* [43] proposed a novel IoT-enabled deep support vector machine (DSVM) and Depthwise separable convolution neural network (DWS-CNN) to classify COVID-19 disease. The DWS-CNN model detects both multiple and binary classes of COVID-19. Gaussian Filtering (GF) was used to preprocess and extract features. The DWS-CNN model is employed for replacing default convolution networks for automatic feature extraction. The diagnostic outcome of the DWS-CNN model is tested using a chest X-ray (CXR) image dataset, and the results are investigated in terms of different performance measures. They have claimed a level of 99.06% and 98.54% accuracy. Waheed *et al.* [44] proposed a novel technique CovidGAN to generate synthetic chest X-ray (CXR) images by using Auxiliary Classifier Generative Adversarial Network (ACGAN). The author claimed that synthetic images generated from CovidGAN could improve the performance of CNNs to detect COVID-19 disease. Their results show an 85% accuracy achieved by using a traditional neural network. After adding synthetic images generated from CovidGAN, the overall accuracy rose to 95%. Pinter *et al.* [45] proposed a hybrid-based model to predict COVID-19. The algorithm integrated a multi-layered perceptron-imperialist competitive algorithm (MLP-ICA) and adaptive network-based fuzzy inference system (ANFIS). This was used to predict the time series of mortality rate and infected individuals. Validation of the proposed method is performed using patient data over 9 days with promising results. Mahanty *et al.* [46] suggested a traditional convolution neural network algorithm for binary classification of pneumonia-based conversion of VGG-19. A decision tree and InceptionV2 are applied over a dataset of CT scan image and X-ray dataset, containing 360 images. The authors claim that the fine-tuned version of the proposed model can achieve training and validation accuracy of 91%. Dansana *et al.* [47] propose research using an exponential model (SIR) and two non-linear growth models (Gompertz, Verhulst) to analyze the coronavirus pandemic across the world. Data used in this research is collected from the John Hopkins University repository over a time span of Jan 30, 2020, to June 4, 2020. The proposed model performs better than the three previous models with an R-score of 0.9981. The summary of related work is presented in table 1.

**TABLE 1:** Summary of Related Work

Ref and year	Author	Data set type	Method	Results			
				ACC	SN	SP	AUC
[24] 2017	Yao et al.	X-rays	DenseNet	–	–	–	79
[25] 2019	Rajaraman et al	X-rays	Modified CNN	96	97	96	99
[26] 2019	Peng et al.	CTs	Markov Random Fields	93	–	–	–
[27] 2019	Baltruschat et al	X-rays	ResNet,	–	–	–	80
[28] 2019	Nasrullah et al	CTs	CMixNet	94	94	91	–
[29] 2019	Stephen et al	X-rays	Customized CNN	93	–	–	–
[30] 2020	Soo Ick et al	clinical photos	ResNet	–	75	80	82
[31] 2020	Lei et al	MRI	3D-DSC	76	–	–	–
[32] 2020	Hashmi et al	X-rays	Weighted Classifier	98	99		
[33] 2020	Jaemin et al	Image dataset	Customized Deep Learning	–	–	–	96
[34] 2020	Khatri et al	X-rays	EMD	83	–	–	–
[10] 2020	Goyal et al	X-rays	VGG16,VGG19, Restnet50	98	99	99	
[35] 2020	Apostolopoulos et al	X-rays	CNN(Transfer learning)	96	98	96	–

## III. Convolutional Neural Network

CNN has shown record performance in several domains such as image classification [48], [49], speech recognition [50], face recognition [51], language translation [52], semantic segmentation [53], image captioning [54], medical image analysis [55], [56], machine translation and other vision tasks. Typically, a CNN consists of convolution, pooling, and dense layers. In the following subsection we explain the building blocks of typical CNN models.

### A. Convolution Layer

The first layer of a CNN is the convolutional layer. It extracts features from an input image with the help of a kernel and produces a feature map (convoluted image) as output. A convolutional operation is composed of several elements including the kernel or filter (kernel matrix), input image (input matrix), and feature map.

### B. Kernel

The kernel is a matrix that is small in relation to the input matrix (input image) and consists of real values. The kernel takes a patch from the input image in specific dimensions (equal to the kernel dimension) and applies a dot operation on the patch and kernel values resulting in a single entry in the feature map. The patch selection is then moved to the right or down-word depending on stride movement. This operation is continued until the whole image is complete; the values of the kernel change after each iteration during training. The final goal is to help the model to achieve the highest accuracy and lowest optimization loss. Therefore, this operation ends-up with learning different features such as edges or color-related features.

### C. Activation Function

Typically, the convolution operation generates linear output. In order to avoid linearity, we use an activation function, which makes the network universal function approximator. Several activation functions are proposed such as sigmoidal, tangent, Rectified Linear Unit (ReLU). Relu is the most widely used as it converts negative values to zero. The mathematical model can be seen in equation 1.

\begin{equation*} f(x) = \max (0,x)\tag{1}\end{equation*}

### D. Pooling

Pooling layers are responsible for reducing the dimensionality of feature maps in a CNN. Several pooling layers are proposed, which include: Max-pooling, Average-pooling, and Sum-pooling. However, Max-pooling showed high performance and widely used for dimensionality reduction. Max-pooling picks the maximum value from the matrix and avoids the rest of the values. The mathematical formulation of Max-pooling is stated in equation 2.

\begin{equation*} pool_{i,j} = \max \limits _{p} f'(x)_{i+p,j+p}\tag{2}\end{equation*}

where $i$ and $j$ represent spatial position.

### E. Fully Connected Layers

Softmax activation is a widely-used activation function for the performance of deep learning approaches. Equation 3 shows the mathematical equation for the softmax function.

\begin{equation*} {Softmax}(x_{i}) = \frac {\exp (x_{i})}{\sum _{j} \exp (x_{j})}\tag{3}\end{equation*}

#### 1) Regularization

Deep neural networks with high learning parameters and lower or noisy training data can suffer from overfitting problems. Overfitting refers to phenomena when the model performs better on training data, but it fails to classify new test instances belonging to the same domain problem. To avoid this issue, we used dropout strategy. During training, the dropout randomly drops neurons with probability $p $ in fully-connected layers. The workflow of the dropout is formulated in equation 4.

\begin{equation*} y_{j} = \sum _{M~\epsilon ~M^{*}} Pr(m)y_{j}^{M}\tag{4}\end{equation*}

In the above equation $y_{j} $ is the expected unit $j $, $M^{*} $ is the set of all thinned network while $y^{M} $ is the output of the unit $M $. $Pr() $ is the probability function in the above equation.

#### 2) Loss Function

The loss or cost function compares the target output and predicted output. It normally minimizes during the training phase. The model is said to be a good learner and near to global minima when its loss value falls to a minimum. We used categorical cross-entropy as the loss function.

## IV. Proposed Methodology

We used a CNN and a GRU on open-access datasets in this research. We obtained three classes of CXR scans from the two sources: COVID-19, Normal, and Pneumonia. We then split the entire dataset into 3 sets: training, validation, and testing. To determine the models’ final classification accuracy, we kept the test split separate (i.e., did not include CXR in the training set). We used a deep convolutional neural network (CNN) for feature extraction while GRU for classification. Figure 3 shows the framework of the proposed method. The methods are stepwise explained in the below sections.

**FIGURE 1.**
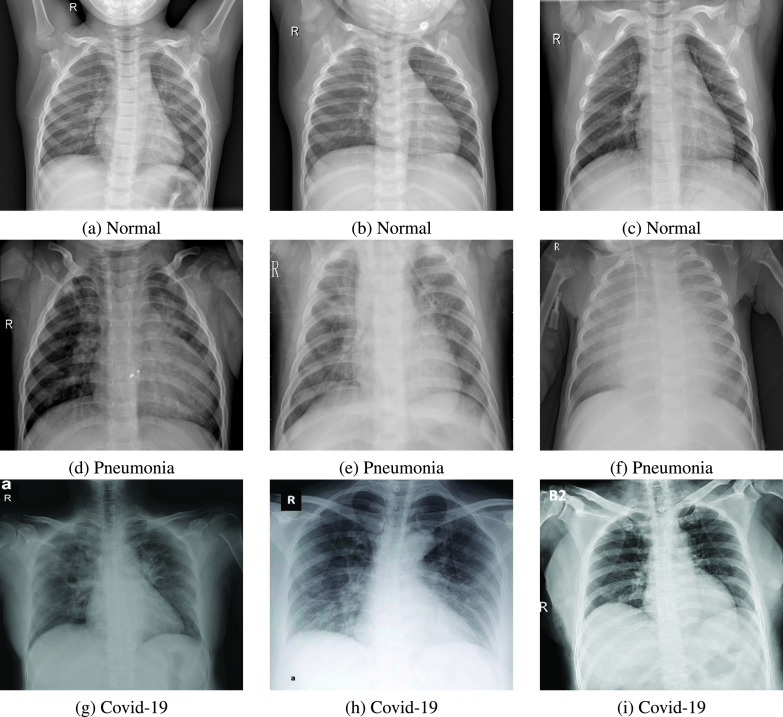
From Top to Bottom row wise: Presents normal, pneumonia and COVID-19 X-ray images.

**FIGURE 2.**
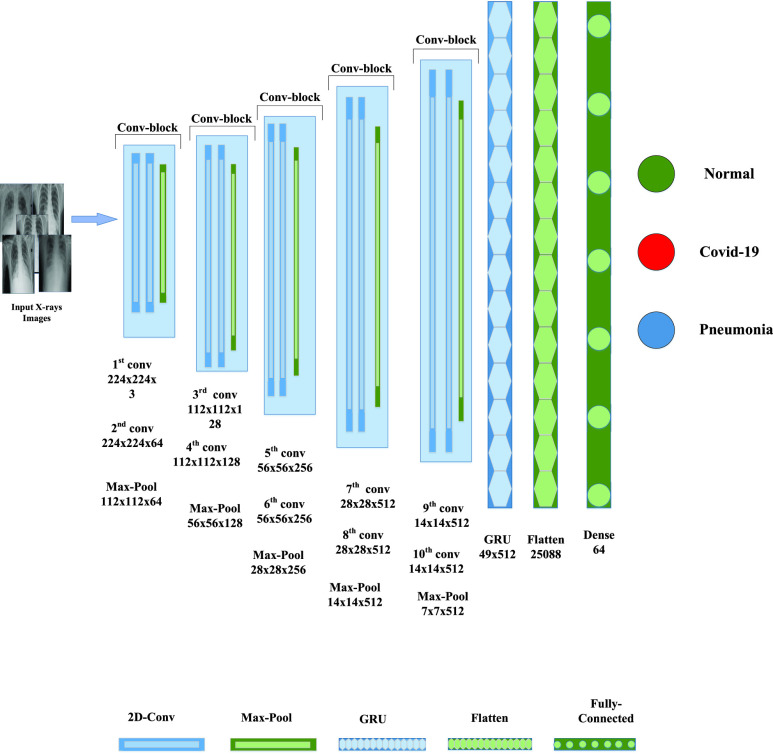
The building blocks of the proposed CNN-GRU model.

**FIGURE 3.**
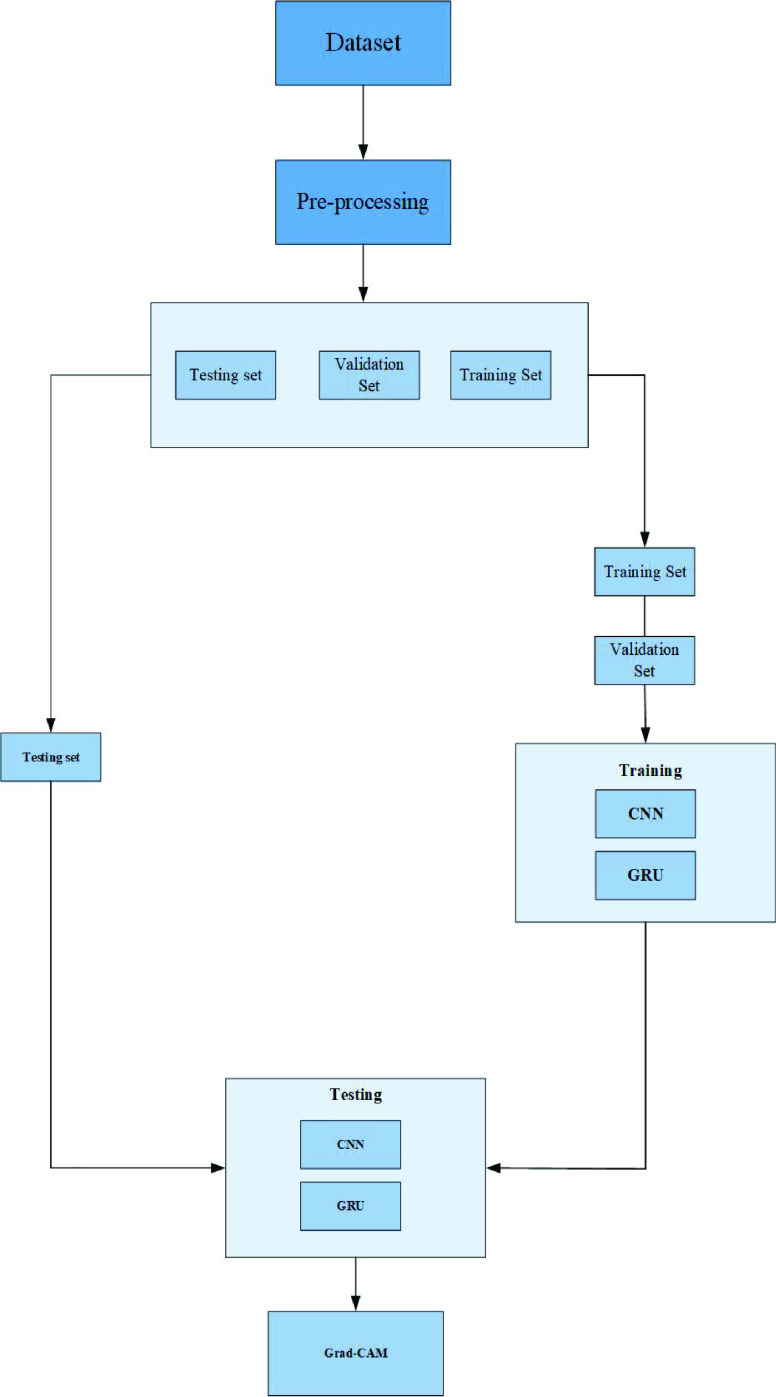
Work flow of the proposed method.

### A. Dataset

The datasets used in this research has been acquired from two different sources. Since COVID-19 is a novel disease, therefore, such datasets are of limited availability for research experiments. In this regard, we approached two different repositories, Joseph Paul Cohen and the Kaggle repository. The acquired dataset from these two repositories consists of 424 X-ray images and three classes. To ensure the balance between classes, we extract an equal number of instances (141) for every class. The dataset is further divided into three sets training, validation, and testing set with the ratio of 70%, 10%, and 20%, respectively. Figure 1 shows the sample of the considered dataset, while table 2 represents the dataset distribution.

**TABLE 2:** Data Distribution

Data Division sets	COVID-19	Normal	Pneumonia	Total
Training	99	98	99	296
Validation	14	15	14	43
Testing	29	28	28	85

#### 1) Pre-Processing

The X-ray images were first passed into the pipeline for preprocessing. In the preprocessing pipeline, data resizing, shuffling, and normalization are performed. The output images were then forwarded to the system for feature extraction.

#### 2) Feature Extraction

Any deep learning model can be easily integrated into our model for feature extraction, but our customized model with defined layers learned best-fit features related to COVID-19 and pneumonia. We also tried added and subtracted the convolutional layers. However, by doing this, our results were then compromised. The figure represents the feature extraction process. Where each image (CXR) is passed through the convolutional layers. The convolutional layer transforms the image into several dimensions (n is the number of defined channels) to feature maps. The first convolutional block receives the input images (X-rays) of 224 $\times $ 224 in three channels (224 $\times $ 224 $\times $ 3). The 224 $\times $ 244 represents the height and width, while 3 represents the image dimensions like Red, Green, Blue (RGB). This block generates 64 feature maps in 122 $\times $ 122 dimensions, further reduced by the max-pooling layer to 112 $\times $ 112 $\times $ 64. Similarly, the second convolutional block receives 122 $\times $ 122 input of dimension 64 and produces the features maps of the dimensions 11 $\times $ 112 $\times $ 128, which is further reduced by a second max-pooling layer 56 $\times $ 56 $\times $ 128. In the same way, these feature maps are passed through $3^{rd}$, $4^{th}$, and $5^{th}$ convolutional blocks. The final feature maps were obtained in the dimensions of 7 $\times $ 7 $\times $ 512, which are further fed to GRU for classification.

#### 3) Gated Recurrent Unit

Typically Deep Neural Networks (DNNs) have the problem of short-term memory. In back-propagation, the gradients may shrink with time, and thus the problem of vanishing gradient occurs. Gradients are values used to update the weights during back-propagation. When the gradient value becomes small, then it may not contribute in learning. Therefore, when a layer in RNN receives a small value gradient, then it may stop learning. To tackle this problem, GRU is the best option. This mechanism can handle the problem of short-term memory. GRU is a simple and new generation of RNN. It consists of two gate reset and update gates. The reset gate is working as a barrier. The decision of keeping or discarding the data is a concern to update the gate, while the reset decides how much previous information should be kept.

#### 4) The Detail Summary of the Model

To better understand the proposed architecture, we have divided our network into several blocks; Conv-block, GRU-block, and FC-block. In total, we have 5 Conv-blocks, 1 GRU-Block, 1 FC-block. Every Conv-block consists of two convolutional layers, while in each block, the last convolutional layer is followed by a max-pooling layer. However, the number of parameters and filters remains disparate in every block.

The output of the last conv-block is then forwarded to GRU-Block to extract time information. To predict the disease, the information is then fed to the last FC-block, which consists of convolutional layers with softmax function. Figure 2 illustrates the architecture of the proposed system.

## V. Performance Evaluation

### A. Experimental Setup

The dataset is split into training validation and testing set with the ratio of 70%, 10%, and 20%, respectively. All the experiments were carried in a Kaggle notebook. The GRU-CNN model is trained and tested using Keras with TensorFlow backend. The experiments were made on maximum epochs 200, with a batch size of 40, and before the softmax classification layer, the dropout layer with 0.5 dropout probability was added. The learning rate is set to 3e-4, while learning rate decay is set to 0.95. The sample code of deep GRU-CNN model is available online at.^1^^1^https://colab.research.google.com/drive/1oyHSleBdz85cH4lyUmfFBGhs_pGgKYI7?usp=sharing

### B. Training and Validation Phase

**FIGURE 4.**
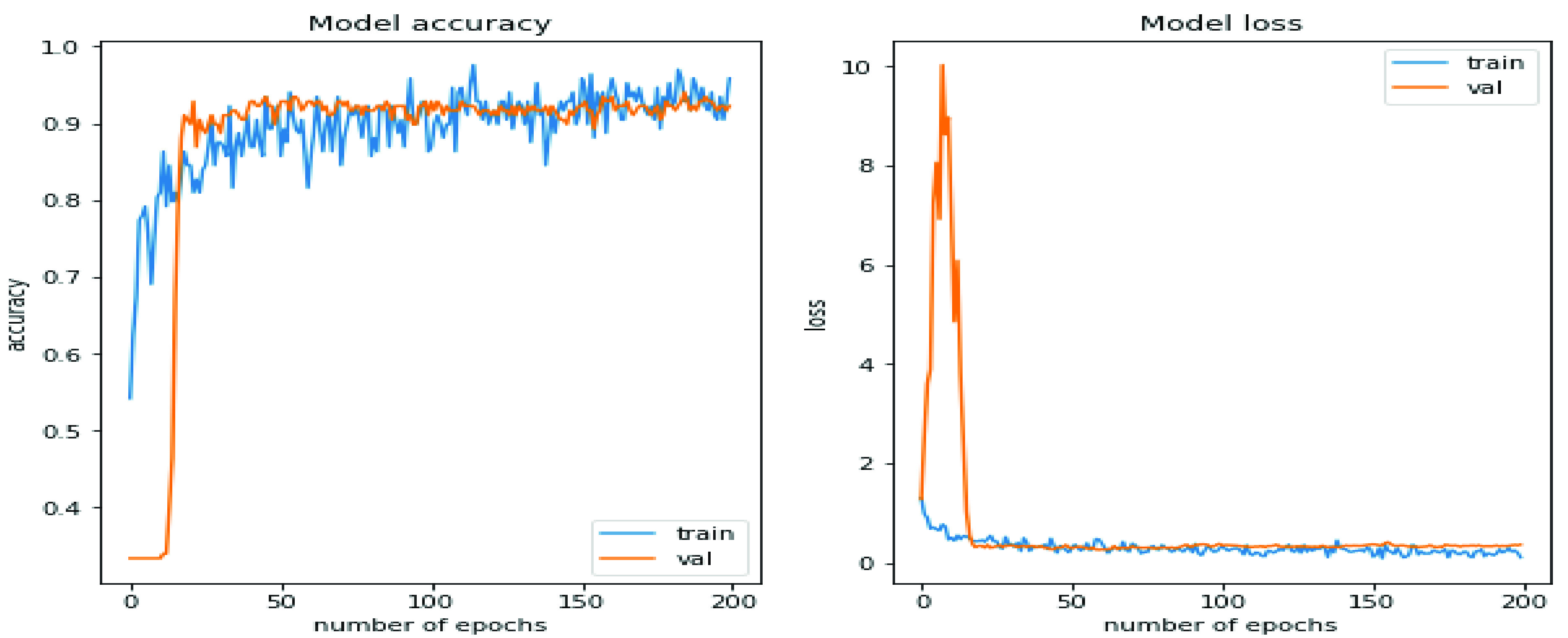
Training accuracy and training loss.

Figure 4 illustrates the proposed model’s performance in the training and validation phase in terms of accuracy and loss. The orange line represents validation accuracy in the model accuracy plot, while the blue line represents training accuracy. Similarly, in the Model loss plot, the training loss is signified by the blue line, whereas validation is presented in the orange line. The obtained training and validation accuracy on the $200^{th}$ epoch is 96% and 93%, respectively. In the same way, training and validation loss on the $200^{th}$ epoch is 0.8 and 0.9, respectively.

**FIGURE 5.**
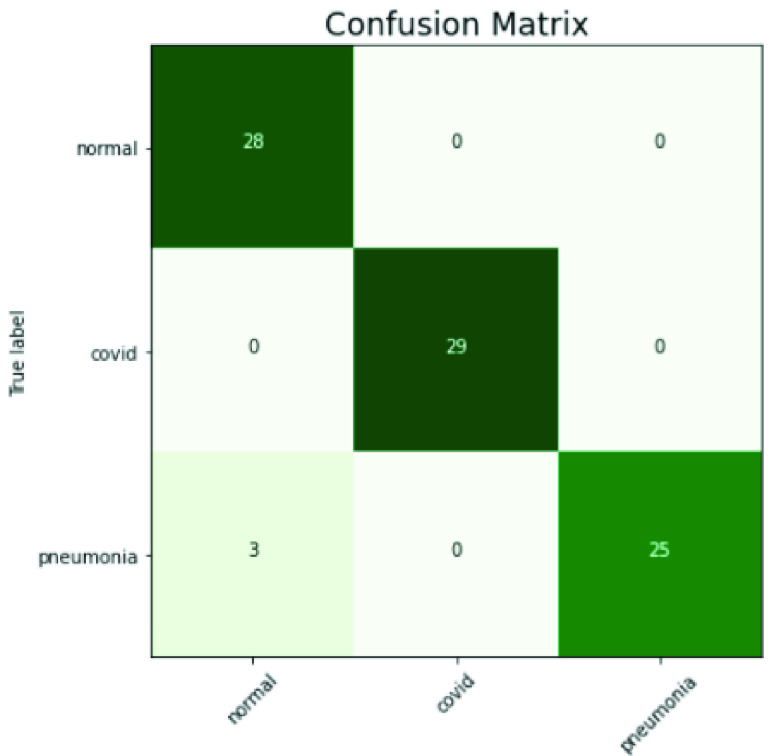
Confusion matrix.

The performance of the proposed model of the test phase is shown in figure 5 in terms of confusion matrix. The first row and column represent instances in normal class, while the second and third show COVID-19 and pneumonia classes. The proposed model classified all the normal and COVID-19 instances correctly. However, in the pneumonia class, among 28 images, only 3 images were miss-classified as normal, whereas the rest of 25 images are correctly classified as pneumonia.

Table 3 shows the precision, recall, and f1-score for each case in the test set. GRU-CNN achieved 0.9 precision 1.00 recall and 0.95 f1-score for normal class. For COVID-19 precision, recall and f1-score are recorded 1.00, 1.00, 1.00, respectively. In the case of pneumonia, the precision, recall, and fi-score are 1.0, 0.89, 0.94, respectively. Among all the scores, the highest score is recorded in the COVID-19 class, while the lowest results values were found in the pneumonia class. The mean score for all the classes are recorded as 0.96, 0.96, and 0.95 in terms of precision, recall and f1-score.

**TABLE 3:** Results of the Proposed CNN-GRU for Individual Normal, COVID-19 and Pneumonia in Terms of Precision, Recall and F1-Score

Labels	Precision	Recall	F1-score	Support
Normal	0.90	1.00	0.95	28
COVID-19	1.00	1.00	1.00	29
Pneumonia	1.00	0.89	0.94	28

**FIGURE 6.**
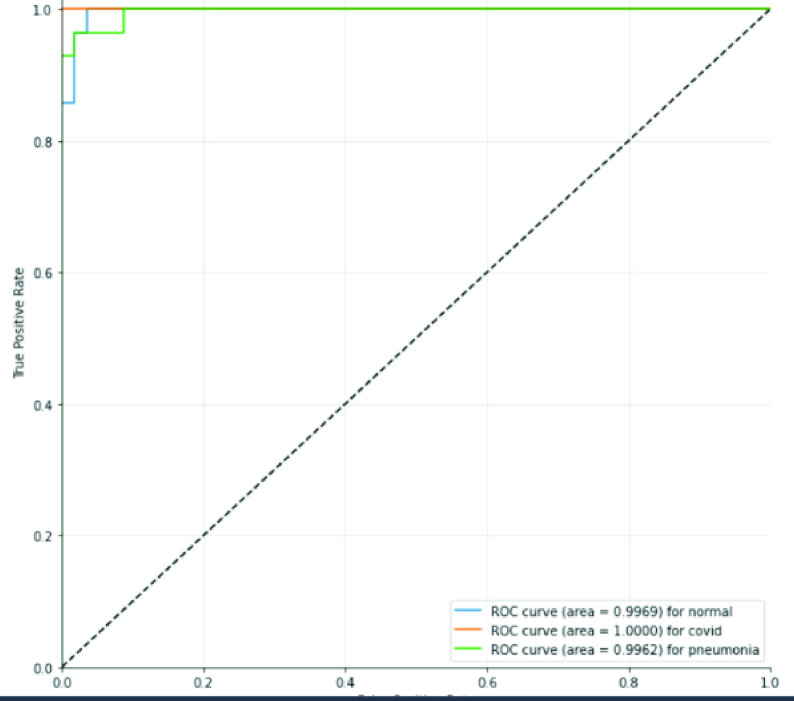
Receiver operating characteristic.

In the same way, Figure 6 represents the ROC curve (receiver operating characteristic curve) for all the classes. It can be seen that class COVID-19 achieved the highest ROC score of 1.00, followed by The Normal class, which is 0.9969. While the class Pneumonia archived the least score of 0.9969. Such results indicate that our model has learned the discriminate features for all the classes.

### C. Grad-Cam

Developing a more robust understanding of deep learning models is an important field of study. Deep Convolution Neural Networks are also referred to as black-box models due to limited knowledge of their internal behavior. An attempt to create more dynamic and explainable deep learning models. Recently, a number of researchers have suggested methods to include class activation maps (CAMs) that represent deep learning predictions with the goal of helping human experts develop intelligible deep learning models. In this regard, the author’s proposed methods to produce gradient-based CAM (i.e., grad-cam) in [57], which highlights the more descriptive input picture relating to the final model prediction for each class. In developing trustworthiness in deep learning-based algorithms, the availability of such information, along with the model’s predictions, plays a vital role. In addition, the existence of the grad-cam enables a human expert (doctor) to verify the efficiency of deep learning.

**FIGURE 7.**
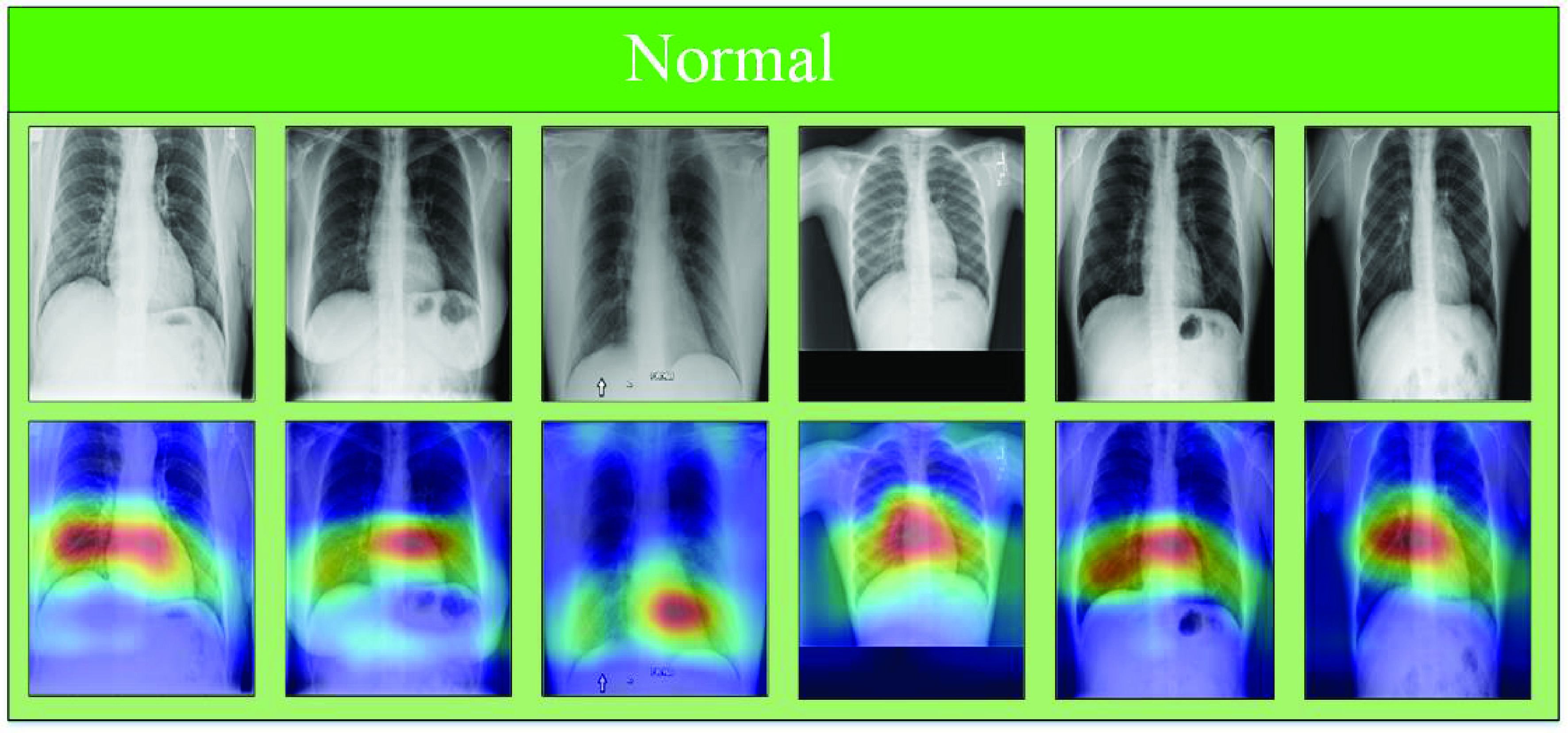
Normal True Negatives: correctly classified normal X-ray scans with corresponding attention maps (CAM). The figure is best viewed in color.

**FIGURE 8.**
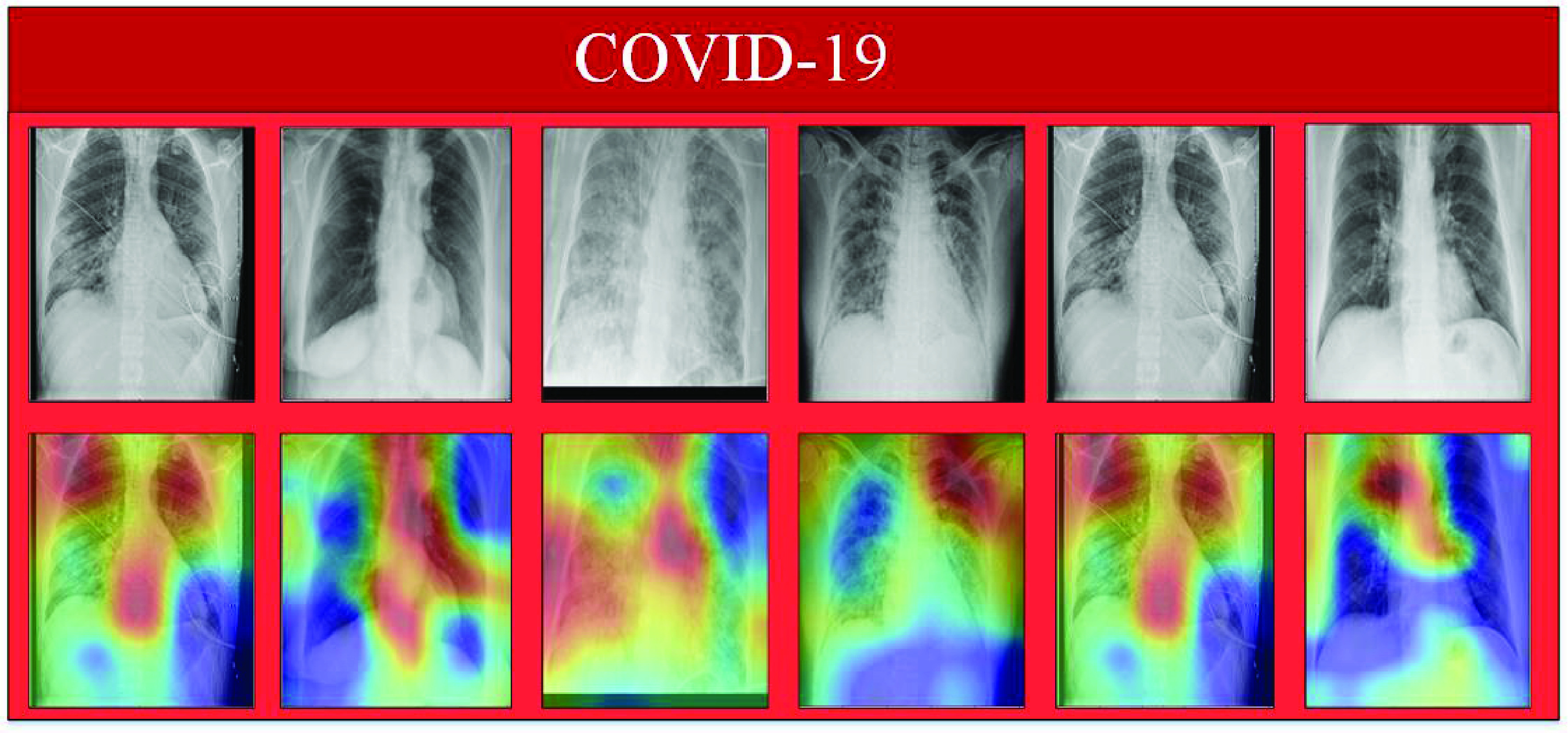
COVID-19 True Positives: correctly classified COVID-19 X-ray scans with corresponding attention maps (CAM). The figure is best viewed in color.

To provide a comparative understanding of the model’s predictions, we also visualize the normal class’s attention maps. The input image, model estimation, and corresponding Grad-Cams of the proposed model, for normal class, are shown in figure 7. In figure 7, the first row represents correctly classified normal X-ray images from the normal class while the second row represents the Grad-Cams against each image. Similarly, in figure 8, 9 shows model Grad-Cams and model prediction for COVID-19 and pneumonia respectively.

**FIGURE 9.**
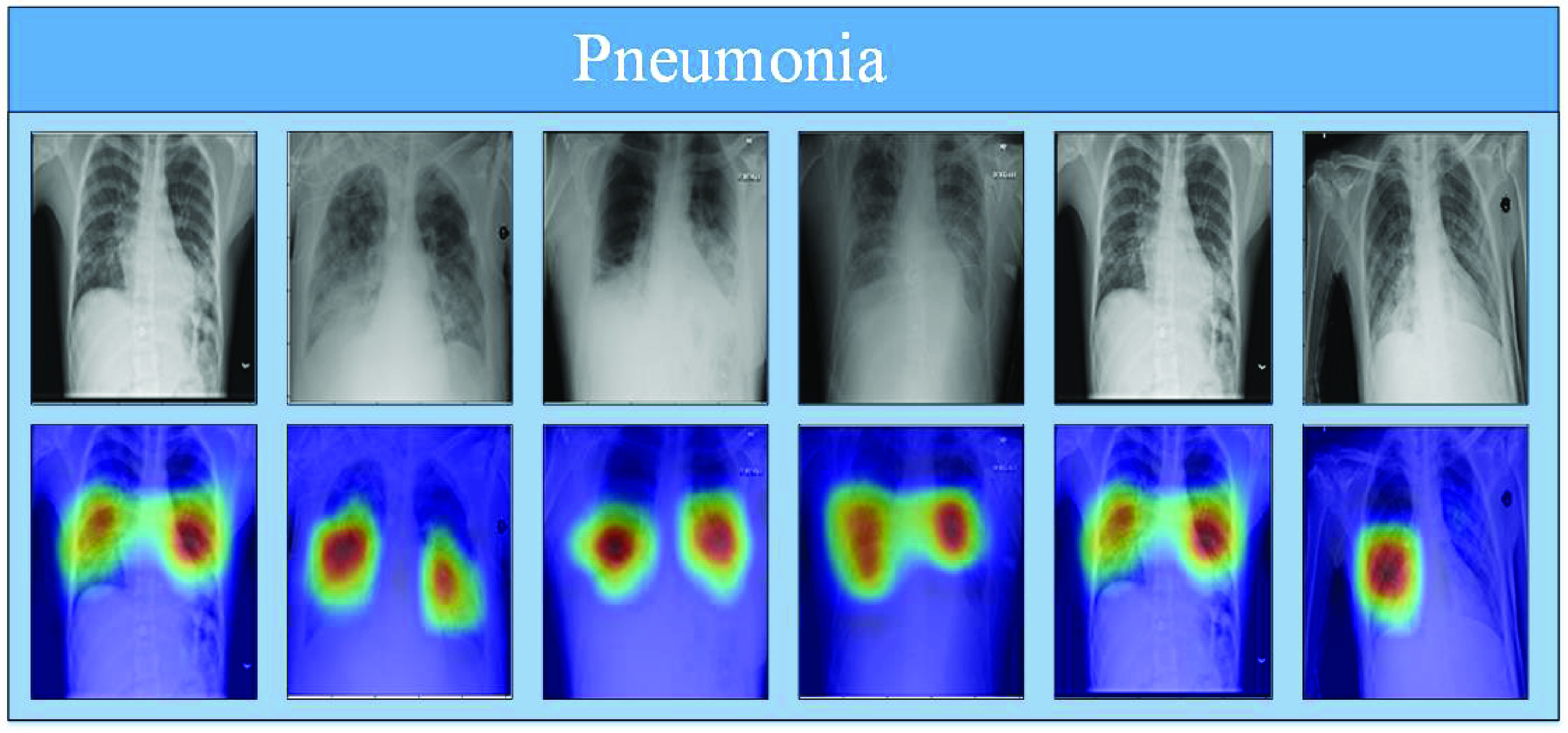
Pneumonia True Positives: correctly classified pneumonia X-ray scans with corresponding attention maps (CAM). The figure is best viewed in color.

The proposed system comes up with limitations like this model is only limited to the X-rays posterior-anterior (PA) view. Therefore, other views of X-rays such as anterior-posterior (AP), lateral and other factors cannot be evaluated. Due to the availability of limited data, our model is trained and tested on minimal data. Lastly, the model performance comparison is only performed with previous algorithms; comparison with human domain experts like radiologists is avoided.

## VI. Conclusion and Future Work

As cases of COVID-19 are rising rapidly, many countries are turned into lockdown and facing a shortage of resources. During this health emergency, it is crucial to detect every single positive case. To overcome the limited testing capacity, we applied a deep GRU-CNN network on the chest X-rays data to detect COVID-19. We used CNN as a feature extractor and GRU as a classifier. By integrating extracted features with GRU, the proposed system’s performance is improved in terms of classification between COVID, pneumonia, and normal instances. In the future, we intend to use Generative adversarial models for data augmentation.
